# Evaluation of CyberKnife® Fiducial Tracking Limitations to Assist Targeting Accuracy: A Phantom Study with Fiducial Displacement

**DOI:** 10.7759/cureus.3523

**Published:** 2018-10-30

**Authors:** Christy Goldsmith, Melanie M Green, Brownwyn Middleton, Ian Cowley, Andrew Robinson, Nicholas P Plowman, Patricia M Price

**Affiliations:** 1 Radiation Oncology, The Cyberknife Centre, HCA Healthcare, London, GBR; 2 Radiation Oncology, Imperial College London, London, GBR; 3 Radiation Oncology, The Harley Street Clinic, HCA Healthcare, London, GBR; 4 Medical Physics, The Harley Street Clinic, HCA Healthcare, London, GBR

**Keywords:** cyberknife, fiducial markers, accuracy, phantom, tumor tracking, target motion, limitations, troubleshooting

## Abstract

Introduction

The underlying assumptions of the CyberKnife® (Accuray, Sunnyvale, CA, US) fiducial tracking system are: i) fiducial positions are accurately detected; ii) inter-fiducial geometry remains consistent (rigid); iii) inter-fiducial geometric array changes are detected and either accommodated with corrections or treatment is interrupted. However: i) soft-tissue targets are deformable & fiducial migration is possible; ii) the accuracy of the tracking system has not previously been examined with fiducial displacement; iii) treatment interruptions may occur due to inter-fiducial geometric changes, but there is little information available to assist subsequent troubleshooting. The purpose of this study was to emulate a clinical target defined with a two, three, or four-fiducial array where one fiducial is displaced to mimic a target deformation or fiducial migration scenario. The objectives: evaluate the fiducial positioning accuracy, array interpretation, & corresponding corrections of the CyberKnife system, with the aim of assisting troubleshooting following fiducial displacement.

Methods

A novel solid-water phantom was constructed with three fixed fiducials (F1,F2,F3) & one moveable fiducial (F4), arranged as if placed to track an imaginary clinical target. Using either two fiducials (F1,F4), different combinations of three fiducials (F1,F2,F4; F1,F3,F4; F2,F3,F4) or four fiducials (F1,F2,F3,F4), repeat experiments were conducted where F4 was displaced inferiorly at 2-mm intervals from 0-16 mm. Data were acquired at each position of F4, including rigid body errors (RBE), fiducial x, y, & z coordinate displacements, six degrees of freedom (DOF) corrections, & robot center-of-mass (COM) translation corrections.

Results

Maximum positioning difference (mean±SD) between the reference and live x, y, & z coordinates for the three fixed fiducials was 0.08±0.30 mm, confirming good accuracy for fixed fiducial registration. For two fiducials (F1,F4), F4 registration was accurate to 14-mm displacement and the F4 x-axis coordinate change was 2.0±0.12 mm with each 2 mm inferior displacement validating the phantom for tracking evaluation. RBE was >5 mm (system threshold) at 6-14 mm F4 displacement: however, F1 was misidentified as the RBE main contributor. Further, F1/F4 false-lock occurred at 16 mm F4 displacement with corresponding RBE <3 mm & COM corrections >13 mm. For combinations of three fiducials, F4 registration was accurate to 10-mm displacement. RBE was >5 mm at 6-16 mm F4 displacement: however, F4 false-lock occurred at 12-16 mm with RBE 5-6 mm. For four fiducials, F4 registration was accurate to 4 mm displacement: however, F4 false-lock occurred at 6-16 mm displacement with concerning RBE <2 & <5 at 6 & 8-mm F4 displacement, respectively. False-locks were easily identified in the phantom but frequently uncorrectable.

Conclusions

Results indicate fiducial positioning accuracy and system output following fiducial displacement depends on the number of fiducials correlated, displacement distance, and clinical thresholds applied. Displacements ≤4 mm were accurately located, but some displacements 6-16 mm were misrepresented, either by erroneous main contributor (two-fiducial array only) or by false-locks and misleading RBE, which underestimated displacement. Operator vigilance and implementation of our practical guidelines based on the study findings may help reduce targeting error and assist troubleshooting in clinical situations.

## Introduction

On-treatment tracking using implanted fiducials for surrogate tumor positioning is commonly applied to soft-tissue targets to overcome X-ray visualization limitations during stereotactic ablative body radiotherapy (SABR) using the CyberKnife® system (Accuray Inc. Sunnyvale, CA, US) [1-2]. Accuray recommends four to six fiducials with specific spacing requirements (see Table 1) and a minimum of three fiducials is required to minimize targeting errors and rotation uncertainty and support corrections with six degrees of freedom (6DOF) [1,3].

**Table 1:** Accuray fiducial placement recommendations for CyberKnife tracking CyberKinfe: Accuray Inc, Sunnyvale, CA, US

Fiducial Placement Recommendations for CyberKnife Tracking:
Implant a minimum of 4 (1 more than required) and a maximum of 6 fiducials
Allow a minimum of 2.00 cm between fiducials
Avoid colinear placement (within the orthogonal imaging plane)
Ensure at least 15-degree angulation between any grouping of 3 fiducials
Ensure a maximum distance of 5–6.00 cm from the lesion
Allow a fiducial "settling" period between implantation and treatment planning

The relative positions of the fiducials to the target and adjacent organs-at-risk (OAR) are established at the planning computed tomography (CT) scan when orthogonal reference digitally reconstructed radiographs (DRR) are generated. Before CyberKnife treatment begins and periodically during treatment (usually every 15-60 seconds) orthogonal live X-ray image pairs are simultaneously acquired by the integrated system and compared with the reference DRR images. 

The CyberKnife fiducial tracking process is often considered the "gold standard," with a reported success rate of >99% [4-5]. Its operation is based on prior knowledge of fiducial size, location, and number. The registration process incorporates band-pass filtering and kernel extraction, the generation of "candidate" fiducial maps, and the calculation of algorithmic "candidates of maximum likelihood." To validate fiducial extraction, the system first matches the x positions of identified fiducials from the two orthogonal images and then relates identified fiducials to the fiducial kernel extracted from the DRR as a confidence measure for the success of the extraction [4]. The system is based on tracking the fiducial array center of mass (COM), assuming inter-fiducial rigid-body geometry, and a rigid body error (RBE) calculation is performed to assess array deformation. If a live fiducial array positioning varies compared with the reference DRR, the system generates a COM translation correction. Before treatment begins, inter-fraction 6DOF COM corrections may be implemented by corresponding couch shifts. During treatment, intra-fraction COM corrections may be automatically implemented by adjustment of the robotic linear accelerator (which has 6DOF capabilities), as small motion uncertainties may be accommodated within applied planning margins (typically 2.00-5.00 mm). The fiducial tracking system may be used alone or in conjunction with motion compensation technologies, such as Synchrony respiratory tracking (Accuray Inc., which synchronizes the 3D translations of the target COM to those of respiration motion, or InTempo for transient random motion in the prostate, which adapts beam delivery to the target COM using time-based imaging [6]. In all cases, if the RBE of the fiducial array is beyond the system safety threshold (RBE ≥ 5.00 mm), COM corrections are not generated and treatment is automatically interrupted. A smaller RBE threshold may be selected by the operator, and/or a threshold for COM may also be applied, which are known as clinical thresholds. These are commonly ~2.00 mm, but their use and size vary with treatment center and/or target site (1.80 mm is currently used at our center for both RBE and COM). When thresholds are breached, beam delivery is automatically withheld whilst treatment radiographers and colleagues troubleshoot the treatment targeting. This may involve implementing a couch-shift correction to realign the patient, or manual investigations and multidisciplinary problem solving may be required prior to implementing appropriate corrections and interventions. Troubleshooting may not be straightforward and there is very little published guidance to assist professionals in determining the best course of action.

During treatment, fiducial displacements may occur, as soft-tissue targets are deformable and undergo inter or intrafraction motion and/or deformation. Interfraction fiducial displacement may also be due to migration, where a fiducial independently moves within tissues to a new anatomical position that is less representative of the target position. Interpretation of fiducial displacement may be difficult, as motion/deformation may not be distinguishable from migration on X-ray imaging. Following a post-implantation "settling" period, fiducial migration is usually considered to be small (≤2.00 mm) and relatively uncommon in most tissues in which CyberKnife fiducial tracking is commonly used (such as the prostate); as such, it is generally considered unlikely to affect tracking accuracy on treatment, with little relevance for intrafraction motion [7-15]. On the other hand, fiducial displacement due to inter and intrafraction target motion and/or deformation may be relatively large. For example, in the prostate, a CyberKnife fiducial tracking study showed intrafraction fiducial array COM shifts that ranged up to 10.00 mm (mean 2.61 mm) with RBE <1.50 mm (when using a clinical threshold of COM ~5.00 mm) [14]. Other prostate radiotherapy studies have reported inter and intrafraction organ points-of-interest, inter-fiducial COM, inter-fiducial distance variations, and single fiducial displacements of 5.0-18.0 mm during some patient treatments [9,12,15-18].

By tracking the fiducial array COM, CyberKnife is considered to have an excellent ability to correct for fiducial array COM offsets and rotations (depending on the number of fiducials used). However, the limits of the CyberKnife fiducial tracking system regarding target array deformations are not well-characterized – even though soft tissue targets are known to be non-rigid and treatment interruptions do occur due to breached RBE thresholds. Other factors known to inhibit fiducial displacement recognition and interpretation in clinical situations include X-ray visualization limitations and the fact that the clinical fiducial array placement may not conform to all recommended ideals. Nonetheless, fiducial array positioning interpretation is critical. In practice, an operator first needs to be notified of any significant fiducial displacement and then needs to determine whether the displaced fiducial represents an unreliable surrogate for a target position that requires the fiducial to be disabled or whether the fiducial is a reliable surrogate for target shape deformation that requires COM corrections to accommodate the new fiducial position — where incorrect interpretation carries the risks of inadequate treatment of the tumor target or increased treatment toxicity due to adjacent OAR irradiation, respectively.

The CyberKnife fiducial tracking system has previously been tested in various ways, including in phantoms with static fiducials [4-5,19-21]. In this study, fiducial displacement was evaluated in a novel phantom model that included fiducial displacement to mimic an on-treatment target deformation or fiducial migration scenario. The consequent impact of fiducial displacement on fiducial registration accuracy, array interpretation, and automated adaptive corrections were investigated for the first time. The main aims were to assess system abilities and limitations following displacements and thereby assist operator troubleshooting.

## Materials and methods

**Figure 1:**
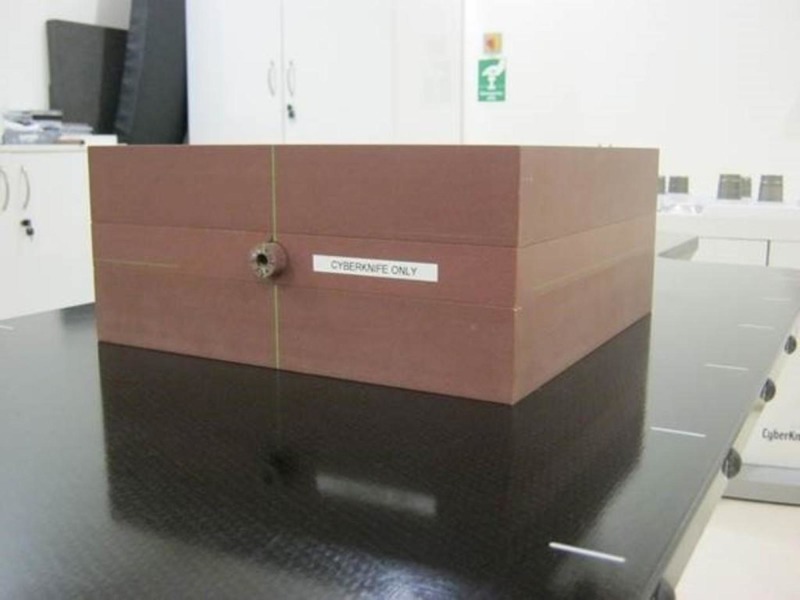
Photograph of experimental phantom in the baseline position

An experimental system was designed to model a clinical target. A solid water 30.00x30.00x3.00 cm phantom block was used that contained three fixed fiducials and one moveable fiducial. The fiducial-bearing phantom was placed horizontally between two further solid water phantom blocks, each 30.00x30.00x5.00 cm, to simulate positioning within the body (Figure 1).

**Figure 2:**
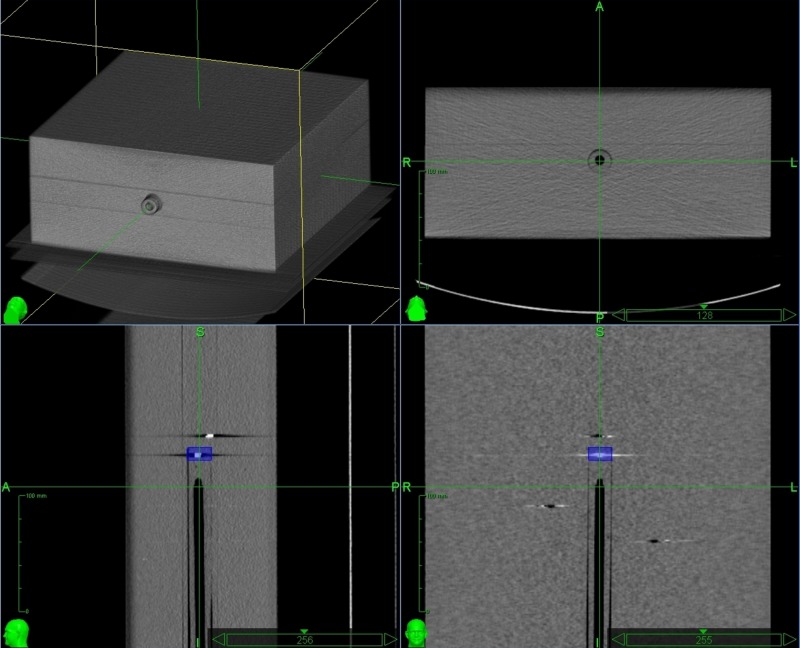
Multi-viewpoint images of the phantom from the MultiPlan system showing the fixed fiducials (F1, F2, and F3) and the migratory fiducial (F4) in the baseline position The blue box in the lower panel images outlines the moveable fiducial F4 at the end of the moveable tube. In the lower right panel, F1 is the fiducial closest to F4, with F2 and F3 shown right and left, respectively. MultiPlan: Accuray Inc, Sunnyvale, CA, US

Within the central fiducial-bearing phantom block, three gold fiducials (CyberMark, Civco Medical Solutions, IA, US), termed F1, F2, and F3, were drilled and glued into fixed positions. A fourth moving fiducial (F4) was similarly fixed into the end of a moveable tube and inserted into a 2.00 cm diameter channel within the central phantom. The vertical and horizontal marks on the tube and the phantom were aligned to assure a reproducible orientation of the tube. When fully inserted into the channel, the tube protruded inferiorly from the phantom by 10.37 mm when measured by digital calipers (Absolute Digimatic Calipers, Mitutoyo UK Ltd, Andover, UK). This was the baseline/CT planning reference position of F4. In this position, the fixed fiducials were arranged in a triangular shape around F4 in the superior and right/left lateral positions (Figure 2) as if arranged around/within a clinical target, e.g. liver lesion. Placement adhered to the recommended Accuray fiducial placement guidelines [21-23] for optimal fiducial positioning (see Table 1).

Prior to CT planning scan acquisition with F4 in the baseline position, the phantom boxes were placed on the CT couch and centrally aligned using in-room lasers, and images were imported into the MultiPlan planning system version 4.5 (Accuray Inc, Sunnyvale, CA, US). An isocentric plan was subsequently created by outlining F4 as a dummy target, purely so the system would generate reference DRRs for a comparative analysis of fiducial positioning.

To ensure a reproducible setup at the start of each experiment, the phantom was arranged on the CyberKnife treatment RoboCouch as per the planning computed tomography (CT) with F4 in the baseline position. A pair of live X-ray images was acquired with the in-room imaging system (120 kV, 100 mA) and the "start" couch corrections were automatically generated and applied to assure accurate and reproducible phantom positioning consistent with the baseline. The default setting of 60% fiducial extraction confidence level (FECL) was used for fiducial registration throughout.

Independent inferior (X-axis) displacement of F4 was simulated by sliding the tube out of the phantom, using digital caliper measurement to increase its protrusion by 2.00 mm. A further pair of live images was acquired and the console information was recorded, including automatically generated x, y, and z coordinate positions of each fiducial, distances between fiducials, 6DOF corrections and RBE values, and the x, y and z COM translation corrections. This procedure was repeated following 2.00 mm incremental shifts of the tube position, up to a total inferior F4 displacement of 16.00 mm. To explore the relationship between fiducial migration and overall translational correction of the system in situations where they may be withheld (i.e., RBE > threshold), an overall COM translational shift analogous to an automated robot correction was calculated at each position of F4 using the root of the sum of the squares formula.

Systematic repeat experiments were conducted to investigate the following fiducial combinations:

· Two fiducials: one fixed and one moving fiducial (F1 and F4)

· Three fiducials: three different combinations each of two fixed fiducials and one moving fiducial (F1, F2, and F4; F1, F3, and F4; and F2, F3, and F4).

· Four fiducials: three fixed and one moving fiducial (F1, F2, F3, and F4).

## Results

Accuracy and validation

Mean (±SD) distances measured between fixed fiducials were F1–F2=103.20±0.05 mm, F1–F3=73.10±0.06 mm and F2–F3=95.70±0.00 mm. Good registration accuracy was observed for fixed fiducials: overall mean x, y, and z positioning differences for fixed fiducials were -0.08±0.23, -0.07±0.30, and -0.01±0.08 mm, respectively; and mean (±SD) positioning differences between reference and live positioning for x, y, and z coordinates for F1, F2, and F3 were -0.17±0.00, 0.17±0.27, and 0.05±0.05 mm; -0.27±0.00, -0.23±0.22, and -0.03±0.00 mm; and 0.20±0.16, -0.14±0.28 and -0.04±0.10 mm, respectively, representing the inherent registration error in defining the center of each fiducial [24]. For the movable fiducial (F4), when F1 and F4 were correlated, mean±SD positioning differences between reference and live positioning in the y and z directions were 0.20±0.21 and -0.19±0.10 mm, respectively, and the mean (±SD) x-axis coordinate shift was 2.00±0.12 mm with each 2.00 mm incremental inferior displacement of F4.

Results summary 

The results obtained for two fiducials, three fiducials, and four fiducials are presented in Table 2.

**Table 2:** Results summary *asterisk denotes erroneous system output based on false-lock. COM = center of mass, RBE = rigid body error

Array	Fiducial Displacement	0 mm	2 mm	4 mm	6 mm	8 mm	10 mm	12 mm	14 mm	16 mm
2-Fiducials	COM (mean±SD) mm	0.00±0.00	0.91±0.02	2.05±0.15	2.94±0.01	3.88±0.01	4.96±0.05	5.93±0.01	6.94±0.05	13.68±0.0*
	RBE (mean±SD) mm	0.01±0.02	1.59±0.16	3.46±0.08	5.23±0.00	6.94±0.00	8.88±0.08	10.65±0.05	12.55±0.13	2.76±0.1*
	False RBE dominant contributor	-	-	-	+	+	+	+	+	+
	False-lock	-	-	-	-	-	-	-	-	+
3-Fiducials	COM (mean±SD) mm	0.00±0.00	0.66±0.09	1.37±0.09	2.39±0.90	2.73±0.11	3.37±0.10	2.13±0.10*	2.13±0.10*	2.13±0.10*
	RBE (mean±SD) mm	0.00±0.00	1.91±0.28	3.63±0.09	5.28±0.12	7.21±0.15	8.78±0.14	5.26±0.59*	5.26±0.00*	5.26±0.59*
	False RBE dominant contributor	-	-	-	-	-	-	-	-	-
	False-lock	-	-	-	-	-	-	+	+	+
4-Fiducials	COM (mean±SD) mm	0.00±0.00	0.50±0.04	1.02±0.00	0.75±0.06*	1.14±0.00*	1.01±0.00*	1.66±0.00*	1.66±0.00*	1.66±0.00*
	RBE (mean±SD) mm	0.64±0.01	2.07±0.18	3.66±0.10	1.88±0.13*	4.21±0.00*	5.29±0.00*	5.60±0.00*	5.60±0.00*	5.60±0.00*
	False RBE dominant contributor	-	-	-	-	-	-	-	-	-
	False-lock	-	-	-	+	+	+	+	+	+

Two fiducials

**Figure 3:**
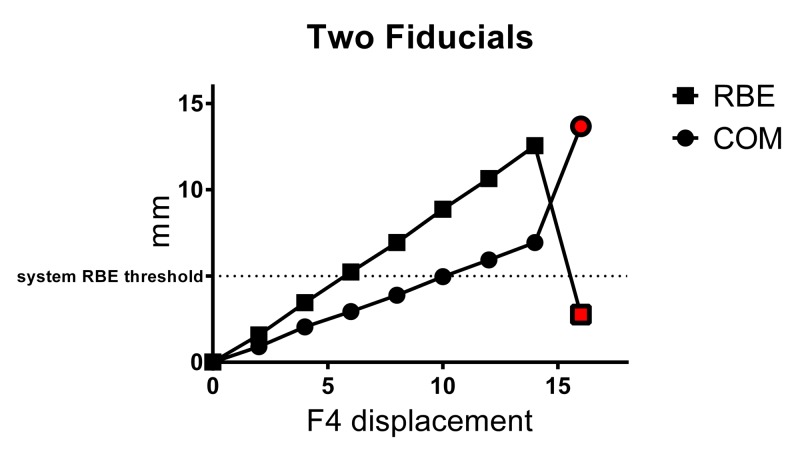
Mean±SD RBE and COM values obtained for two-fiducial array with displacement of one fiducial (F4) Red symbols represent values based on false-locks. RBE = rigid body error, COM = center-of-mass translation

For a single suboptimal two-fiducial array comprising F1 and F4, the mean COM translational shifts and mean RBE values obtained with a two-fiducial array, for each 2.00 mm x-axis displacement of F4, are illustrated in Figure 3 and presented in the results summary table above. When two fiducials were correlated (F1 and F4), accurate fiducial registration was achieved from a 0.00–14.00 mm F4 displacement. Up to 14.00 mm, RBE increased with a linear positive relationship to F4 displacement. As expected, due to the three-dimensional (3D) spatial location of F1 and F4, the magnitude of RBE did not exactly correlate with the magnitude of F4 x-axis displacement. COM translations were also linearly related to fiducial displacement and both COM and RBE values obtained showed excellent reproducibility with repeat experiments (SD≤0.16 mm). Notably, COM translations obtained for two fiducials were larger when compared with those obtained for three and four fiducials. At 4.00 mm F4 displacement, RBE and COM translations were 3.46±0.08 and 2.05±0.15 mm, respectively. In a clinical situation, this may result in the treatment pause, depending on clinical thresholds applied at treating centers. RBE values were >5.00 mm (system threshold) at 6.00–14.00 mm F4 displacement, which would result in automatic treatment interruption to permit operator investigations and interventions, with COM translations withheld. However, at 6.00–14.00 mm F4 displacement, F1 was erroneously identified as the RBE main contributor (indicated by a red square on the operator console) on three of three occasions, which could hinder the interpretation of fiducial positioning without operator inspection of treatment images and comparison with anatomical reference points. At 16.00 mm F4 displacement, the system did not correctly detect the position of F1 and/or F4 (one or more false-locks occurred with standard 60% FECL) and gave an erroneous RBE of 2.76±0.12 and COM translations of 13.68±0.02 mm. A false-lock is not identified by the system, although the true fiducial positions were clearly visible in the homogeneous phantom to the investigators. In a clinical setting, this situation would result in treatment pause if typical clinical thresholds of ~2.00 mm were applied; however, the RBE value may be misleading, as it indicates a relatively small geometric array deformation and any subsequent recognition of the false-lock would be operator and image dependent. Notably, the false-lock occurred between the baseline position (where F4 was "expected" to be located) and the actual position of F4, i.e. the displacement was underestimated. Furthermore, operator intervention to correct the false-lock – by identifying the correct fiducial position and helping the system to locate the fiducial position by minimizing the tracking range in which the system looked for candidate fiducials – was not very successful, as the system was resistant to locking onto the true fiducial position. Operator intervention to correct fiducial localization only resolved the situation on one of three occasions using this method.

Three fiducials

**Figure 4:**
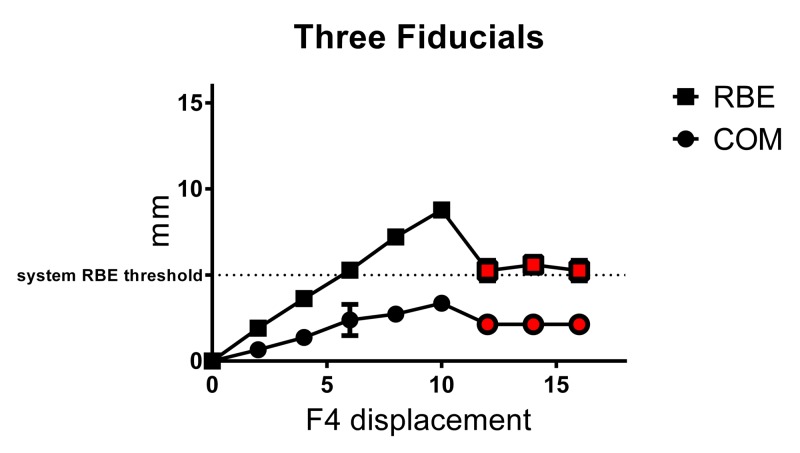
Mean±SD RBE and COM values obtained for three-fiducial array with displacement of one fiducial (F4) Red symbols represent values based on false-lock. RBE = rigid body error, COM = center-of-mass translation

The mean COM translation shifts and mean RBE values obtained with three different three-fiducial arrays, for each 2.00 mm x-axis displacement of F4, are illustrated in Figure 4 and presented in the results summary table above. When three fiducials were correlated, accurate fiducial registration was achieved at 0.00–10.00 mm F4 displacement for all three fiducial combinations investigated (F1, F2, and F4; F1, F3, and F4; and F2, F3, and F4). Up to 10.00 mm, RBE values increased linearly with a positive relationship to F4 displacement, similar to results for two fiducials. COM translations also increased linearly up to 10.00 mm F4 displacement but, as expected, were smaller than those obtained with two fiducials. Notably, both COM translations and RBE values showed greater variation for pooled data (SD≤0.9 mm and 0.6 mm, respectively) compared with values obtained for the two-fiducial array, indicating that specific fiducial positioning of the array combination affects system output to some degree. Similar to results obtained with two fiducials, at 6.00–16.00 mm F4 displacement, RBE values were >5.0 mm system threshold, which would result in treatment being withheld. At 4.00 mm F4 displacement, mean COM and RBE were 1.37±0.09 and 3.63±0.09 mm respectively, which may result in treatment interruption, depending on clinical thresholds employed. When F4 was displaced 12.00–16.00 mm, the system did not correctly detect the position of F4 (F4 false-lock), resulting in erroneous RBE of 5–6 mm and mean COM 2.13±0.10, which would result in treatment interruption in a clinical situation. As before, the false-lock position fell short of the actual fiducial position and again was resistant to correction by minimizing the tracking range.

Four fiducials

**Figure 5:**
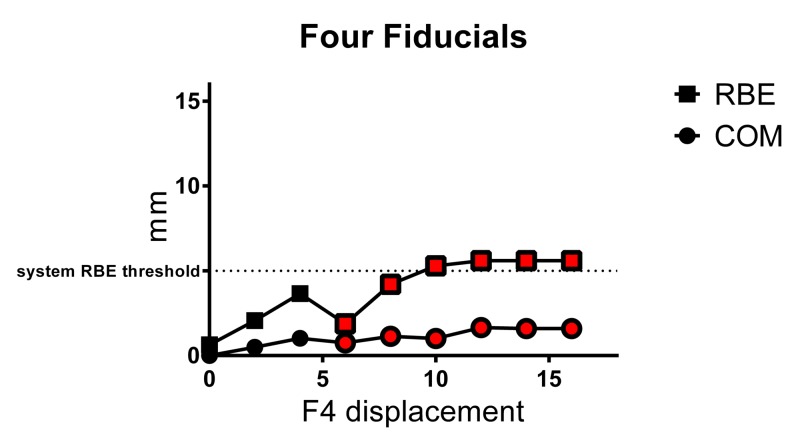
Mean±SD RBE and COM values for four-fiducial array with displacement of one fiducial (F4) Red symbols represent values based on false-lock. RBE = rigid body error, COM = center-of-mass translation

The calculated mean COM translation shifts and mean RBE values obtained for a single four-fiducial array (F1,F2, F3, and F4), for each 2.00 mm X-axis displacement of F4, are illustrated in Figure 5 and presented in the results summary table above. When four fiducials were correlated, accurate fiducial registration was achieved at 0.00–4.00 mm F4 displacement. Up to 4.00 mm, RBE values increased linearly with a positive relationship to F4 displacement distance. COM translational values also increased linearly up to 4.00 mm F4 displacement but were smaller than those obtained with two or three fiducials. Both RBE and COM values showed little variation with repeat experiments (SD≤0.18 and ≤0.06 mm, respectively). However, when F4 was displaced 6.00–16.00 mm, F4 was not correctly identified and false-lock occurred, resulting in erroneous COM corrections that were <2.00 mm. Moreover, from 2.00–8.00 mm F4 displacement, RBE ranged between 1.88–4.21 mm, where 9.00 mm F4 displacement gave RBE <5.00 mm (below system threshold), and at 6.00 mm F4 displacement, both RBE and COM were <2.00 mm (i.e. below typical RBE and COM clinical thresholds). In a clinical situation, treatment interruption may, therefore, depend on clinical thresholds used. Notably, an RBE clinical threshold of 1.8 mm may enable treatment interruption in these scenarios.

Additional observations

An overall pattern for COM translations was observed following the displacement of one fiducial, whereby the COM translational correction approximated the migration distance of F4 (mm) divided by the total number of fiducials correlated, i.e. shift correction ≈ migration distance/number of fiducials. However, this pattern begins to break down when false-locks occurred and is unreliable from 10.00 mm F4 displacement. A further analysis of RBE values showed an overall mean±SD RBE between fixed fiducials F1 and F2, F2 and F3, and F1 and F3 were 0.24±0.02, 0.23±0.13, and 0.58±0.24 mm, respectively, with overall range 0.19–0.70 mm, showing low inherent registration variation for fixed fiducials. Maximum RBE values were smaller than the fiducial displacement distance in all cases, illustrating that because of relative fiducial positioning, RBE magnitude is a distinct measure to (and may typically underrepresent) fiducial displacement distance. In addition, when pooled RBE values obtained between fiducial pairs F1 and F4, F2 and F4, and F3 and F4 were examined with 0.00–16.00 mm F4 displacement distance (ignoring values obtained for false-locks), RBE values between F3 and F4 were consistently smaller compared with F1 and F4 and F2 and F4, indicating relative fiducial positioning affects the RBE values generated.

Summary

If the phantom study results are extrapolated to clinical situations, the following points are notable.

• For arrays of two, three, and four fiducials, all displacements of one fiducial ≤ 4.00 mm were accurately detected.

• Displacement of one fiducial ≤2.00 mm resulted in RBE and COM values that would not breach commonly used 2.00–5.00 mm RBE and COM clinical thresholds, indicating that they would not result in treatment pause but would likely be accommodated as a small target deformation with an appropriate COM adjustment.

• Displacement of one fiducial by 4.00 mm resulted in RBE <5.00 mm, indicating that whether treatment interruption occurred would depend on clinical thresholds employed. To ensure automatic treatment pause with the displacement of one fiducial ≥4.00 mm for arrays of two, three, and four fiducials, the minimum RBE clinical thresholds indicated to be necessary are 2.76, 3.63, and 1.88 mm, respectively.

• For larger displacements of one fiducial (>4.00 mm), the two-fiducial array showed better tracking results compared with the four-fiducial array. The two-fiducial array showed a greater range of detection accuracy following F4 displacement (from 0.00–14.00 mm vs 0.00–4.00 mm), and thus increased displacement distance before false-lock occurred (16.00 mm vs 6.00 mm). In addition, RBE values that would induce treatment interruption due to RBE system threshold breach occurred at a lower displacement distance (6.00 mm vs 10.00 mm).

• For many instances where displacements would definitely result in treatment pause due to system threshold breach (i.e. RBE >5.00), the system output information is either erroneous (i.e. false RBE main contributor or false-lock) and/or would involve relatively large COM robot translation corrections that are notably greater than typical margins applied for motion uncertainties, thereby increasing risk if there is array interpretation uncertainty, which may make operator interpretation and troubleshooting difficult.

## Discussion

Overall, the results showed good accuracy for fixed fiducial localization, with low inherent variation. With the displacement of one fiducial in one direction (to mimic a target deformation or fiducial migration scenario), the total number of fiducials in an array, the displacement distance, and the clinical COM/RBE thresholds applied were found to determine system response.

Smaller displacements of one fiducial of 0.00–4.00 mm showed good fiducial registration accuracy for arrays of two, three, or four fiducials, with reassuring, approximately representative RBE and appropriate compensatory COM corrections. The results additionally indicate that in a clinical situation, small displacements of ≤2.00 mm are likely to be accommodated within typical planning margins as a target deformation, whereas displacements of 4.00 mm may either be accommodated as a target deformation or may lead to treatment interruption for operator investigation, depending on the clinical thresholds employed.

However, system limitations were shown for larger fiducial displacements of 6.00–16.00 mm. The accuracy of fiducial positioning and thus reported console information was found to depend on the number of fiducials correlated in the array and displacement distance, with accuracy affected by the occurrence of false RBE main contributor (two-fiducial array only) and the incidence of false-locks that underestimated fiducial displacement and gave misleading RBE and COM (two-, three-, and four-fiducial arrays). When a false-lock occurred, the reported RBE did not accurately reflect the displacement and, in some instances, despite fiducial displacement >5.0 mm, resulted in RBE that was less than the 5.00 mm RBE system threshold. This suggests that treatment may not be interrupted following larger displacements unless sufficiently small clinical thresholds are used. Furthermore, tracking range correction limitations were experienced when the investigators attempted to help the system recognize the true position of the displaced fiducial. All of these factors may inhibit the recognition, interpretation, and appropriate correction of fiducial displacement in vivo. This is because a false-lock is not reported on the operator console and, similar to the identification of a true RBE main contributor, may not be identifiable without a close inspection of treatment and planning images and a comparison with anatomical landmarks, where image clarity may additionally be limited in a clinical situation. Thus, without sufficiently small clinical thresholds and operator vigilance, it is possible that a false-lock may go unnoticed or may be recognized despite misleading information and be considered to represent a deformation but may not be correctable. Furthermore, as false-locks were shown for larger displacements that resulted in RBE <5.00 mm (as occurred with a 6.00 or 8.00 mm displacement of one fiducial when using a four-fiducial array), it raises the possibility that larger fiducial displacements (e.g. due to intrafraction target deformation) may occur more frequently in clinical situations than may be accurately detected by the tracking system.

The main limitations of the study were of the phantom model system. The first was that the study was wholly conducted in a phantom and, as is the case with all model/phantom studies, the results may only be extrapolated/inferred to clinical situations. The second is that only the specific geometry relating to the phantom model was studied. This was intended to model a "typical" clinical scenario, with placement and sizing similar to a moderate liver lesion. Although a total of five baseline geometries were investigated, each with nine different positions of the moveable fiducials, and it is notable that three different combinations of three fiducials gave very similar results, it is possible that other relative positions of fiducials may give different results. The third limitation is that the fiducials were in a homogenous solid water phantom, and the y/z axis positions of all fiducials were as expected throughout. This does not accurately reflect a clinical situation, as fiducial registration and positioning are unlikely to be as accurate in vivo due to concurrent y and z-axis alterations or the displacement of more than one fiducial, leading to overall 3D array orientation changes. Other important factors, including less soft-tissue contrast, multiple tissue densities in the region of interest, deformation of adjacent tissue, and X-ray attenuation, are also likely to inhibit fiducial positioning in a clinical situation [6,19-23]. It is, therefore, likely to be much harder for the system or indeed treatment radiographers, to accurately identify and interpret fiducial array positioning in vivo following fiducial displacement, which, in turn, infers that the occurrence of false RBE main contributor and false-locks may well be greater in clinical situations.

Many of the study findings were reassuring. First, the sub-millimeter level of tracking accuracy obtained for fixed fiducials and for smaller fiducial displacements ≤4.0 mm confirms the fiducial registration accuracy shown in previous studies [19,21,24]. Second, the good agreement between fiducial localization and caliper measurements validates the phantom for fiducial tracking evaluation. Third, for fiducial displacements ≤2.00 mm, for two, three, or four-fiducial arrays, the system accurately tracked fiducials and adjusted treatment with automatic corrections that could be accommodated within typical treatment margins (assuming fiducials were located within the target volume), thus minimizing the risk of targeting error. Fourth, smaller COM corrections were obtained when using more fiducials, and fifth, tracking with three or four fiducials did not result in "false RBE main contributor" scenarios that may significantly impact troubleshooting and array interpretation. The latter findings support recommendations for a minimum of three fiducials to minimize targeting errors and enable 6DOF optimal corrections [1-3]. It is additionally reassuring to note that the system output tends towards not overtreating motion uncertainties for larger fiducial displacements when more fiducials are used, as potential targeting inaccuracies (COM adjustments) were smaller with more fiducials and false-locks underestimated displacement. Thus, even though false-locks occurred more readily with larger displacements in an array with more fiducials—and regardless of whether the displacement was based on false-lock or a subsequent interpretation of fiducial displacement is migration or deformation or whether correct or incorrect—overall, the study findings showed a tendency of the system towards either undertreatment or minimization of over-treatment for larger fiducial displacements when more fiducials are used.

For larger displacements (>4.00 mm), some findings showed system limitations may be considered counterintuitive or unexpected or were contrary to other data, indicating that a four-fiducial array improves tracking accuracy and interpretation [20-21]. First, we found that fiducial registration was more accurate over greater distances with fewer fiducials and, conversely, false-lock occurred more readily with four fiducials at smaller displacement distances. Although this appears to be in contrast to a prior phantom study that investigated the registration of fixed fiducials with purposefully degraded image quality and showed fewer false-locks with more fiducials [20], we postulate that it is the same inherent characteristics of the fiducial registration process that underlies both sets of results, i.e. more fiducials allow greater certainty and 3D localization accuracy of where a particular fiducial "should" be located using rigid geometry assumptions (as the system is inherently designed to track a rigid body). With a rigid fiducial array, more fiducials, therefore, show fewer false-locks, but if a fiducial has moved from the original position, the result will be the opposite with an increased likelihood of false-lock. Usefully, as outlined in Table 3, the increased fiducial localization accuracy with fewer fiducials could be exploited by operators where there is uncertainty over fiducial positioning or potential false-lock, as the temporary deselection of fiducials in an array may be a helpful strategy to increase fiducial registration accuracy between specific fiducial pairs (acknowledged anecdotally as a helpful strategy among operators). Second, importantly, we found that displacements ≥4.0 mm may not necessarily lead to treatment interruption, as it depends on: i) the exact RBE and COM generated, ii) the RBE and COM thresholds applied, and iii) whether the RBE and COM values obtained are based on false-lock. For example, with four fiducials, we found that a 6.00 mm F4 displacement resulted in a false-lock that reported an erroneously low COM and RBE: if this occurred in a clinical situation with typical 2.00 mm COM and RBE clinical thresholds applied, treatment would not be paused. The displacement may not be recognized without operator vigilance and, instead, may be accommodated as a small target deformation with automated COM corrections. Similarly, the results indicate that treatment interruption may not occur with 4.00 mm displacement of one fiducial when employing a two, three, or four-fiducial array unless a pre-set COM clinical threshold of <2.00 mm and an RBE clinical threshold of <3.46 mm are used; an 8.00 mm displacement of one fiducial when employing a four-fiducial array, unless an RBE clinical threshold of <4.21 mm is used; and a 16.00 mm displacement of one fiducial when employing a two-fiducial array, unless a COM clinical threshold <13.68 mm and an RBE clinical threshold <2.76 mm are used. Third, when using a three- or four-fiducial array, the maximum RBE that was obtained was 5.60 mm, indicating that the maximum RBE output reaches a plateau — despite a considerable displacement of one fiducial up to 16.00 mm and regardless of true or false-lock—which may be misleading for true fiducial positioning. Fourth, we found that when larger displacements would lead to treatment interruption, operator interpretation of fiducial positioning may be affected because of the potential occurrence of false RBE main contributor and false-locks or by concerns of the relatively large COM corrections if the fiducial position is accepted as a reliable target surrogate. Fifth, a three-fiducial array appeared to be more reliable than a four-fiducial array, in terms of whether treatment would be interrupted with accurate information that would support operator interpretation when one fiducial was displaced up to 10.00 mm. Lastly, we found false-locks were difficult to correct and infer that they may be difficult to identify in vivo, as an operator would not know a false-lock had occurred unless the true fiducial position could be clearly seen in treatment images. As with a previous study, we additionally found false-locks may be associated with a high fiducial extraction confidence limit (FECL) [20], which may impede their recognition. Further, we found that even if a false-lock is clearly identified, it may not necessarily be correctable.

**Table 3:** Recommended practices and troubleshooting guidelines COM = center of mass, RBE = rigid body error, FECL = fiducial extraction confidence limit

Summary Recommendations
Beneficial practices when using CyberKnife® treatments with fiducial tracking:
Follow Accuray recommendations for 4-6 fiducials (Table 1) to allow optimal on-treatment rigid-body tracking.
Use the clinical threshold for both COM translational corrections AND RBE to enable treatment interruption for fiducial displacement >4.00 mm that may occur due to target deformation or fiducial migration and may result in false-lock. Our results indicate a clinical RBE threshold of 1.80 mm and COM of 2.00 mm or less may help treatment interruption even with false-locks; however, subsequent recognition of false-lock would be operator dependent.
Be aware that fiducial displacements >4.00 mm may occur due to deformation or migration but may not interrupt treatment without sufficiently tight tracking parameters and, when treatment is interrupted, may not be easily recognizable.
Be vigilant for false-locks; wherever possible, visually check treatment images.
Accuray recommends at least three fiducials to allow rotation corrections (Table 1). If a two-fiducial sub-optimal array is used, be skeptical of RBE main contributor "red-square" guidance and check the positioning using anatomical landmarks.
Recommendations to improve array interpretation and decision-making when RBE/COM thresholds are breached:
Verify fiducial localization positioning with anatomical landmarks, if possible, to determine fiducial array positioning compared with planning. If available, use cone-beam imaging to compare fiducial positioning and anatomy on the day of treatment.
Consider and check for possible false-locks or false RBE main contributor. Be aware that false-locks may occur due to displacement, may be difficult to identify, tend to underestimate the displacement, and may be associated with a high FECL [20].
Try the temporary deselection of fiducials to allow a systematic RBE assessment of fiducial pairs, as it may be helpful to increase fiducial registration accuracy and so identify true fiducial positioning/false-lock situations; however, a false-lock may not necessarily be correctable by reducing the tracking range.
Assess the displacement cause and potential targeting error risk. Consider whether the displacement is inter or intrafraction or due to target deformation or migration, and estimate the risk if array interpretation is wrong, where COM corrections and, therefore, targeting error, approximate the migration distance/number of fiducials.
If displacements >4.00 mm are considered due to genuine target deformation, consider increasing clinical thresholds to better accommodate shape changes.

The study findings have several important implications. They indicate that although tracking range accuracy is excellent for single fiducial displacements ≤2.00 mm, which appear to be typically accommodated as a target deformation, there is reduced tracking range accuracy for larger displacements >4.0 mm. These are likely conferred by the rigid body assumptions and positional verification with DRR positioning that underlie the systems fiducial registration processes. The accuracy of fiducial registration following displacement is affected by a number of factors, including the number of fiducials correlated and fiducial displacement distance: X-ray discrimination, FECL, and sampling rate are additional factors [14,20,24-25]. As previously outlined, because fiducial localization is likely to be much harder in clinical situations, it is possible that false RBE main contributor and false-lock situations due to larger 6.0–16.0 mm fiducial displacements may occur more frequently in clinical situations than may be detected and/or corrected. It is additionally notable that because i) false-locks tend to underestimate displacement and may not be recognized, ii) manual tracking correction limitations prevent the adequate correction of false-locks, and iii) operators may be reluctant to accept recognized large displacements as deformations due to targeting error risk, larger displacements that may occur due to true target deformation may be undertreated. As prior CyberKnife fiducial tracking studies to examine tumor motion have relied on the assumption that fiducial registration is accurate and without limitations, it is additionally possible that the incidence and size of fiducial displacements in vivomay be greater than previously measured in CyberKnife fiducial tracking studies. These implications may have particular relevance for on-treatment deformations. For example, in prostate SABR, studies using other methods have shown relatively large inter and intrafraction prostate point deformations, increases in inter-fiducial distances, or fiducial marker displacements of 5.00–18.00 mm [9-10,13,15,17-18,26-27]. Yet, the range of motion reported with the CyberKnife fiducial tracking system is generally smaller and may be restricted by fiducial registration accuracy limitations, rigid-body assumptions, and applied clinical thresholds [11,14,16]. Similarly, in the liver, a magnetic resonance imaging (MRI) study in healthy volunteers showed an average range of deformation up to 7.1 mm [28], whereas a CyberKnife tumor study reported little deformation with a mean fiducial displacement of 0.5 (range 0–3.0) mm [29].

Based on our results and their potential implications for clinical situations, we have developed recommendations and troubleshooting guidelines, as given in Table 3.

## Conclusions

CyberKnife fiducial tracking is often considered the "gold standard" in the performance evaluation of other registration algorithms. In this novel experimental phantom model with one, two, or three fixed fiducials and one migrating fiducial, we found that the imaging of fiducial position by the in-room imaging system was reassuringly highly accurate (to within 0.24 mm) and reproducible (SD<0.09mm), as long as fiducials were correctly identified.

Although acknowledged to be limited, the study indicated that the accuracy of fiducial positioning, corresponding console information, and likelihood of treatment interruption depended on the number of fiducials correlated in an array, fiducial displacement distance, and clinical thresholds used. Rigid fiducials and smaller (<4.00 mm) single fiducial displacements were consistently detected and appropriately reported when using an array of two, three, or four fiducials. However, larger (≥ 4.00 mm) displacements of one fiducial were not always accurately detected or reported. This was due to either false RBE main contributor information (with two fiducials) or because of false-locks that underestimated displacement, gave corresponding misleading RBE, and were resistant to correction (where false-locks unexpectedly occurred more readily when using an array of four fiducials as compared with two fiducials). When extrapolated to clinical situations, the results indicate that without operator vigilance and sufficiently tight pre-set clinical parameters, displacements of 6.00–16.00 mm that may occur in vivo due to target deformation or fiducial migration may not lead to treatment interruption and may not be recognized or adequately interpreted and corrected following treatment interruption. Strategies to mitigate system limitations, such as optimizing fiducial arrays, frequent visual inspection of treatment images, careful selection of treatment parameters, and operator vigilance, are, therefore, recommended for optimal targeting, particularly for target structures subject to larger on-treatment deformations. The implementation of our troubleshooting guidance based on the study findings, including temporary fiducial deselection to inspect tracking accuracy, may additionally assist array interpretation and decision-making when issues arise with fiducial tracking in clinical situations.

## References

[1] Fasola CE, Wang L, Adler JR, Soltys SG, Gibbs IC, Koong AC, Chang DT (2015). CyberKnife. Principles and Practice of Stereotactic Radiosurgery.

[2] Dieterich S, Gibbs IC (2011). The CyberKnife in clinical use: current roles, future expectations. IMRT, IGRT, SBRT. Advances in the Treatment Planning and Delivery of Radiotherapy.

[3] Benedict SH, Yenice KM, Followill D (2010). Stereotactic body radiation therapy: the report of AAPM task group 101. Med Phys.

[4] Hatipoglu S, Mu Z, Fu D, Kudufalli G (2007). Evaluation of a robust fiducial tracking algorithm for image-guided radiosurgery. Proc SPIE 6509 Medical Imaging: Visualization and Image-Guided Procedures.

[5] Penney GP, Weese J, Little JA, Desmedt P, Hill DL, Hawkes DJ (1998). A comparison of similarity measures for use in 2-D-3-D medical image registration. IEEE Trans Med Imaging.

[6] Accuray Inc (2018). Accuray Inc. CyberKnife technical specifications: CyberKnife robotic radiosurgery. http://cyberknifelatin.com/pdf/brochure-tecnico.pdf.

[7] Delouya G, Carrier J-F, Béliveau-Nadeau Béliveau-Nadeau, D D, Donath D, Taussky D (2010). Migration of intraprostatic fiducial markers and its influence on the matching quality in external beam radiation therapy for prostate cancer. Radiother Oncol.

[8] Kumar KA, Wu T, Tonlaar N, Stepaniak C, Yenice KM, Liauw SL (2013). Image-guided radiation therapy for prostate cancer: a computed tomography based assessment of fiducial marker migration between placement and 7 days. Pract Radiat Oncol.

[9] Crook JM, Raymond Y, Salhani D, Yang H, Esche B (1995). Prostate motion during standard radiotherapy as assessed by fiducial markers. Radiother Oncol.

[10] Kupelian PA, Willoughby TR, Meeks SL, Forbes A, Wagner T, Maach M, Langen KM (2005). Intraprostatic fiducials for localization of the prostate gland: monitoring intermarker distances during radiation therapy to test for marker stability. Int J Radiat Oncol Biol Phys.

[11] Lei S, Piel N, Oermann EK (2011). Six-dimensional correction of intra-fractional prostate motion with CyberKnife stereotactic body radiation therapy. Front Oncol.

[12] Moreau J, Biau J, Achard JL (2017). Intraprostatic fiducials compared with bony anatomy and skin marks for image-guided radiation therapy of prostate cancer. Cureus.

[13] Poggi MM, Gant DA, Sewchand W, Warlick WB (2003). Marker seed migration in prostate localization. Int J Radiat Oncol Biol Phys.

[14] Xie Y, Djajaputra D, King CR, Hossain S, Ma L, Xing L (2008). Intrafractional motion of the prostate during hypofractionated radiotherapy. Int J Radiat Oncol Biol Phys.

[15] Schallenkamp JM, Herman MG, Kruse JJ, Pisansky TM (2005). Prostate position relative to pelvic bony anatomy based on intraprostatic gold markers and electronic portal imaging. Int J Radiat Oncol Biol Phys.

[16] Kupelian P, Willoughby T, Mahadevan A (2007). Multi-institutional clinical experience with the Calypso system in localization and continuous, real-time monitoring of the prostate gland during external radiotherapy. Int J Radiat Oncol Biol Phys.

[17] Fung AYC, Ayyangar KM, Djajaputra D, Nehru RM, Enke CA (2006). Ultrasound-based guidance of intensity-modulated radiation therapy. Med Dosim.

[18] Hegde J, Cao M, Yu V, Kishan A, Shaverdian N, Lamb J, Steinberg M (2018). Magnetic resonance imaging guidance mitigates the effects of intrafraction prostate motion during stereotactic body radiotherapy for prostate cancer. Cureus.

[19] Jung J, Song SY, Yoon SM (2015). Verification of accuracy of Cyberknife tumor-tracking radiation therapy using patient-specific lung phantoms. Int J Radiat Oncol Biol Phys.

[20] Subedi G, Karasick T, Grimm J (2015). Factors that may determine the targeting accuracy of image-guided radiosurgery. Med Phys.

[21] Murphy MJ (2002). Fiducial-based targeting accuracy for external-beam radiotherapy. Med Phys.

[22] Kee ST (2018). Fiducial placement to facilitate the treatment of pancreas and liver lesion with the CyberKnife system. Accuray Incorporated.

[23] Dieterich S (2005). Dynamic tracking of moving tumours in stereotactic radiosurgery. Robotic Radiosurgery.

[24] Kitamura K, Shirato H, Shimizu S (2002). Registration accuracy and possible migration of internal fiducial gold marker implanted in prostate and liver treated with real-time tumor-tracking radiation therapy (RTRT). Radiother Oncol.

[25] Antypas C, Pantelis E (2008). Performance evaluation of a Cyberknife G4 image-guided robotic stereotactic radiosurgery system. Phys Med Biol.

[26] Ghilezan MJ, Jaffray DA, Siewerdsen JH (2005). Prostate gland motion assessed with cine-magnetic resonance imaging (cine-MRI). Int J Radiat Oncol Biol Phys.

[27] Padhani AR, Khoo VS, Suckling J, Husband JE, Leach MO, Dearnaley DP (1999). Evaluating the effect of rectal distension and rectal movement on prostate gland position using cine-MRI. Int J Radiat Oncol Biol Phys.

[28] von Siebenthal M, Szekely G, Lomax AJ, Cattin PC (2007). Systematic errors in respiratory gating due to intrafraction deformations of the liver. Med Phys.

[29] Xu Q, Hanna G, Grimm J (2014). Quantifying rigid and nonrigid motion of liver tumors during stereotactic body radiation therapy. Int J Radiat Oncol Biol Phys.

